# A Temporal Model of Human IgE and IgG Antibody Function

**DOI:** 10.3389/fimmu.2013.00235

**Published:** 2013-08-09

**Authors:** Andrew M. Collins, Katherine J. L. Jackson

**Affiliations:** ^1^School of Biotechnology and Biomolecular Sciences, University of New South Wales, Sydney, NSW, Australia

**Keywords:** IgG subclasses, humoral immunity, class switching, affinity maturation, IgE, antibody function, B cell differentiation

## Abstract

The diversity of the human antibody repertoire that is generated by V(D)J gene rearrangement is extended by nine constant region genes that give antibodies their complex array of effector functions. The application of high throughput sequencing to the study of V(D)J gene rearrangements has led to significant recent advances in our understanding of the antigen-binding repertoire. In contrast, our understanding of antibody function has changed little, and mystery still surrounds the existence of four distinctive IgG subclasses. Recent observations from murine models and from human studies of VDJ somatic point mutations suggest that the timing of emergence of cells from the germinal center may vary as a consequence of class switching. This should lead to predictable differences in affinity between isotypes. These differences, and varying abilities of the isotypes to fix complement and bind FcRs, could help coordinate the humoral defenses over the time course of a response. We therefore propose a Temporal Model of human IgE and IgG function in which early emergence of IgE sensitizes sentinel mast cells while switching to IgG3 recruits FcγR-mediated functions to the early response. IgG1 then emerges as the major effector of antigen clearance, and subsequently IgG2 competes with IgG1 to produce immune complexes that slow the inflammatory drive. Persisting antigen may finally stimulate high affinity IgG4 that outcompetes other isotypes and can terminate IgG1/FcγR-mediated activation via the inhibitory FcγRIIB. In this way, IgG antibodies of different subclasses, at different concentrations and with sometimes opposing functions deliver cohesive, protective immune function.

It is almost 50 years since the complete set of human antibody isotypes was first described (1). For over 30 years, associations have been explored between antibody classes and subclasses and the response to particular pathogens (2). And for almost 30 years, the relationships between cytokine production and antibody class switching have been reported (3). Other rich sources of data that have guided thinking about antibody isotype function have been studies of immunodeficiencies, and the disease susceptibilities with which they are associated (2, 4). Yet despite literally thousands of such studies, and despite significant insights into the particularities of humoral immunity, no proposal has emerged that describes how IgG antibody subclasses and other antibody isotypes *work together* to provide protective immune functions. Here we propose a Temporal Model of human IgE and IgG antibody function, in which there is a programed order to the emergence of the different IgG isotypes that reflects their genomic organization, with switching and emergence being promoted or delayed at different critical points through the action of cytokines. We suggest that early in the germinal center reaction, IgM^+^ B cells switch to both IgE and IgG3. Subsequently, IgG1 cells switch and emerge, followed by IgG2-committed cells and finally, if antigen persists, by IgG4-producing cells.

The Temporal Model has its genesis in recent observations of IgE-switched cells in the mouse. These studies suggest that the IgE response is not usually a late development arising from an expanded clone of IgG-committed cells that develops through the germinal center reaction. Rather, it has been shown that IgE class-switched murine cells usually develop and exit the germinal center reaction in the early phase of an immune response, and that they rapidly differentiate into plasmablasts and plasma cells (5, 6). The IgE-secreting plasma cells carry fewer somatic point mutations in their rearranged V(D)J genes than IgG-secreting plasma cells (6), and as a consequence their secreted antibodies are likely to be of lower affinity.

There can be no doubt that IgE antibodies can also be produced late in a response. Recent studies have confirmed the existence of high affinity IgE, and of sequential switching to IgE within the germinal centers of mice (7, 8). No attempt has been made here to incorporate such late IgE into the model. The functions of secretory IgA in mucosal secretions and of serum IgA are also not considered, but the temporal model provides a coherent view of the separate and joint activities of early IgE and the IgG subclasses.

Reports of early IgE in murine models provide a new perspective from which to consider some unusual features of human IgE antibody gene sequences. We have shown that IgE-associated VDJ genes from non-allergic individuals carry very few somatic point mutations, and some IgE sequences carry no mutations at all (9). In individuals with atopic dermatitis, unmutated sequences have also been seen at relatively high frequency (10). In parasitized individuals, we have seen more highly mutated IgE sequences (11), but these sequences did not carry the pattern of mutations that is considered the mutational signature of antigen selection within the germinal center reaction (12). In some, though not all allergic conditions, IgE sequences also lack this pattern of mutation (9, 10).

These studies can be understood if IgE class switching in humans, as in the mouse, can occur early in the germinal center reaction, and if such switching is rapidly followed by the differentiation of IgE-switched cells into plasmablasts that leave the germinal centers. Some continuing accumulation of somatic point mutations might then take place, outside the germinal centers (13). This would give the mutations in those IgE sequences a distinctly different pattern to that which is seen in IgG sequences that emerge after multiple rounds of selection within the germinal centers. Such selection typically leads to an accumulation of non-synonymous (replacement) mutations in the complementarity determining regions of the antibody genes (12).

In the context of invasion by pathogens, the production of early IgE antibodies could allow widely dispersed mast cells to function as sentinel cells (14), alerting the immune system to further incursions or spread of the pathogens. Early IgE could function in this way, despite its low affinity, because low affinity IgE has been shown to function well on the surface of mast cells and basophils, if it is directed against multiple epitopes on multivalent antigen (15, 16).

If class switching to IgE is rapidly followed by departure of cells from the germinal center, the possibility that switching to other isotypes may lead cells to follow other distinct developmental pathways cannot be ignored. We have therefore reconsidered the functions of human IgG subclasses, and this has been done in the light of our observations of somatic point mutations in antibodies of different IgG subclasses. These observations provide the broadest possible overview of humoral immunity. In an analysis of almost 1,000 VDJ genes isolated from people living in an area of endemic parasitism, a surprising and statistically significant relationship was seen (11). IgG3-associated VDJ genes were the least mutated VDJ gene sequences, and the mean number of mutations seen in sequences associated with the other subclasses corresponded to the position of each constant region gene within the IGH gene locus. That is, IgG3 < IgG1 < IgG2 < IgG4.

We hypothesize that differences in mean levels of mutation arise because human B cells tend to follow a programed sequence of class switching and departure from the germinal center reaction. We propose that cells first switch from IgM to IgG3, then to IgG1 and to IgG2 and finally to IgG4 following the genomic ordering of the constant region genes (Figure 1). This is not to deny the reality of alternative switch pathways under the influence of particular cytokines (17). We propose that class switching is driven by underlying probabilities, and switching is linked to emergence from the germinal centers, leading to the generalizable sequence of the Temporal Model. Through changes in probabilities associated with the expression of adhesion molecules and chemokine receptors, switching could be closely followed by emergence, or emergence could follow variable periods of proliferation, mutation, and selection within the germinal centers. The model does not attempt to resolve the timing of these events for each isotype.

**Figure 1:**
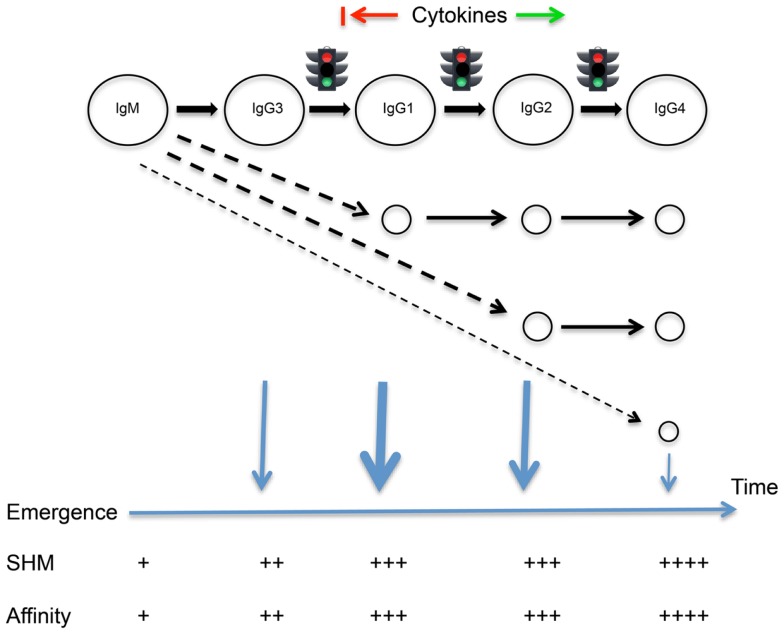
**The Temporal Model of IgE/IgG class switching and departure from the germinal center reaction**. A programed sequence of sequential switching is highlighted, though alternative switch pathways are also indicated. The timing of switching events and the emergence of different cell types from the germinal center reaction is suggested by the extent of somatic point mutations carried by the cells. Many cytokines may act to promote or delay class switching, as indicated.

The Temporal Model has parallels with models of division-linked phenotypic change, including class switching, which suggest that predictable order can emerge from stochastic processes because of differences in the underlying probabilities of different outcomes (18, 19). It also is in line with modeling of the dynamics of murine division-linked isotype switching that suggested that the outcome of isotype switching, under the indirect influence of cytokines, is biased toward switching to the immediate downstream neighboring constant region gene (20).

Though a simple relationship between mutation numbers and affinity in any sequence cannot be assumed, accumulating mutations are generally considered to give rise to higher affinity antibodies through selection within the germinal centers (21). Sequential departure of cells from the germinal centers should therefore ensure that antibodies of different isotypes have predictable differences in affinity. This in turn should ensure that despite the different isotypes having some opposing actions, and despite the changing relative concentrations of the different isotypes over time (22), all antibodies at the time of their production should be able to play their assigned roles. It should also ensure that inflammatory processes are tightly controlled, through the temporal coordination of antibodies that have striking differences in their abilities to bind FcγR and to fix complement.

There is good evidence that the IgG3 response occurs early, is relatively transient and is of relatively low affinity (22, 23). This is supported by our sequencing study, for IgG3-associated VDJ genes had the fewest mutations of the IgG subclasses (mean 17.7 mutations), 31% of the sequences had less than 10 mutations and 7% of the sequences had no mutations at all (11). We propose that class switching to IgG3, the first IgG subclass gene in the human IGHC locus, first brings beneficial FcγR-mediated defenses into play. The accumulation of some somatic point mutations during the differentiation of IgG3-committed cells should ensure that most IgG3 antibodies have experienced some affinity maturation, and the specific physicochemical properties of IgG3 should mean that the switch from IgM to IgG3 does not lead to a crippling loss of binding avidity.

The principal “early” antibody, IgM, is able to provide useful protection despite its low affinity, because of the multivalent nature of secreted IgM, and because of its flexibility (24). The long hinge region of IgG3 makes it the most flexible human IgG antibody (25). This should facilitate bivalent binding of high avidity to repeated determinants on the surface of an invading pathogen. As part of the early response, IgG3 antibodies would have to work with IgM antibodies to efficiently trigger complement fixation and engagement with FcγR-bearing cells. In fact, IgG3 has the highest affinity of the IgG subclasses for C1q, the first component of the classical complement cascade (26). It also has the highest affinity for the FcγRIIIA and FcγRIIIB receptors, and its affinity for FcγRIIA is second only to IgG1 (27).

The elongated hinge region makes IgG3 vulnerable to catabolism. IgG3 has a half-life of just 7 days (28) and shares a short half-life with IgM (∼5 days) (29) and IgE (∼3 days) (30). This rapid turnover of all three kinds of “early antibody” should facilitate the ever-increasing dominance, as a response progresses, of higher affinity antibodies of other isotypes.

In our study of VDJ rearrangements, IgG1-associated sequences were significantly more mutated than IgG3 sequences. The mean mutation of VDJ utilizing the IgG1 gene, positioned immediately downstream from IgG3, was 21.0 somatic point mutations, and only 13% of sequences had fewer than 10 mutations (11). We therefore suggest that IgG1-committed cells are the next cell type to differentiate and depart the germinal centers. Although having on average just three more mutations than IgG3 sequences, we suggest that a number of days are likely to separate the average time of departure of IgG3-committed cells and IgG1-committed cells. It is generally accepted that mutations accumulate at the rate of about one mutation per cell division (31), and centroblast division time is thought to be around 7 h (32). It is likely, however, that as increasing numbers of mutations accumulate, the probability that further random mutations are beneficial is low (33). The speed with which selected sequences accumulate mutations is therefore likely to slow over the course of a response.

Class switching to IgG1 leads to the secretion of more highly mutated, complement-fixing, FcγR-binding IgG antibodies that often dominate the response to bacterial and viral invaders (34, 35). Certainly, IgG1 antibodies are the most abundant serum antibodies (36). With their shorter hinge regions, IgG1 molecules lack flexibility but with their higher affinity for antigen, even monovalent binding should be stable and effective. And with their high affinity for C1q (26) and FcγRI, FcγRII, and FcγRIII (27), such IgG1 antibodies would continue driving inflammatory processes and antigen clearance.

Many studies report that IgG1 antibodies appear relatively early in the immune response, and in fact IgG1 and IgG3 are often the only IgG subclasses detected in a response (37, 38). This could result from early antigen clearance preventing the appearance of IgG2 and IgG4 antibodies. It could also reflect delays in downstream class switching as a result of the prevailing cytokine milieu. T cell cytokines are often said to drive class switching to IgG1 and IgG3 (39). Alternatively, they could be said to delay class switching to IgG2 and IgG4, by their promotion of the IgG1 response.

IgG2 antibodies are the second most abundant serum antibodies and the IgG2 gene is positioned immediately downstream of IgG1. IgG2 antibodies are seen at concentrations that are comparable to IgG1 antibodies, and that are much higher than the typical serum concentrations of IgG3 and IgG4. In contrast to IgG1 antibodies, IgG2 antibodies fix complement very poorly (40, 41) and interact very weakly with FcγR (27). In our sequence study, IgG2 antibodies carried a higher mean number of mutations (22.0) than IgG1 antibodies (21.0) (11). It is difficult to believe that this higher level of mean mutations would lead to biologically significant differences in mean antibody affinity, but certainly IgG2 antibodies must share high affinity with IgG1 antibodies. All these features of the humoral response require explanation. In particular, any model of IgG subclass function must explain how IgG1 and IgG2 antibodies, the two most abundant antibody isotypes, work together to deliver protective immunity despite their diametrically opposed properties.

We hypothesize that IgG2-committed cells emerge from the germinal center reaction, on average, shortly after the development of the IgG1 response. We further hypothesize that IgG2 functions as an anti-inflammatory “partner” to more inflammatory IgG1 antibodies, “dampening down” the inflammatory response by its competition with the IgG1 isotype (Figure 2). Working together, IgG1 and IgG2 antibodies could provide a spectrum of activity, from the highly inflammatory “pure IgG1 response,” to the “pure IgG2 response” that results in immune complexes that cannot interact with FcγR-bearing cells or with molecules of the complement system.

**Figure 2:**
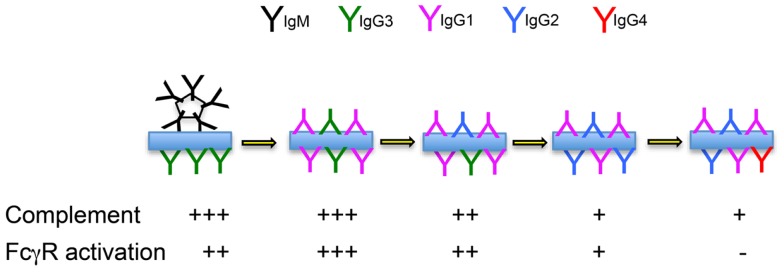
**Immune complex formation over the course of a prolonged immune response**. The likely complement-fixing and FcγR-binding abilities of immune complexes formed by antibodies of mixed isotypes are indicated.

Mutation data suggests that IgG1 and IgG2 antibodies have similar affinities. If this is the case, they will compete on a level playing field, where antibody concentrations prevail. We propose that the relative concentrations of IgG1 and IgG2 are the result of the balance of cytokines that either promote or delay switching to IgG2. These proportions will be seen in the immune complexes that form during the response, and the outcome of varying proportions of IgG1 and IgG2 antibodies will be immune complexes having varying avidity for complement and for FcγR. IgG2 can therefore be conceptualized as an anti-inflammatory brake on the inflammatory actions of IgG1. In certain circumstances, switching may occur quickly, leading to a response that is dominated by IgG2. This could help explain reports that IgG2 dominates the antibody response to carbohydrates response (42). In fact similar concentrations of IgG1 and IgG2 antibodies have often been reported in response to carbohydrate antigens (43–45), and IgG2 antibodies are also a conspicuous part of the response to many protein antigens (46). It is clear that the chemistry of carbohydrate antigens cannot explain the IgG2 response.

The human IgG4 response is often described as an anti-inflammatory blocking response (47), but the apparent functions of these antibodies have been difficult to reconcile with their very low concentrations. We believe that their mode of action is revealed by the levels of mutations that are seen in IgG4-associated VDJ sequences. IgG4 is the most distal of the IgG subclass genes within the heavy chain constant region locus. In our sequence study, IgG4 antibodies carried the highest mean number of VDJ mutations of the IgG subclasses (mean: 27.1), and no unmutated IgG4-associated VDJ sequences were seen (11). This suggests to us that IgG4-committed cells are (typically) the last cell type to emerge from the germinal center reaction. They are therefore likely to be the highest affinity antibodies. This is indirectly supported by the circumstances in which IgG4 antibodies are conspicuous. Serum IgG4 concentrations are elevated in chronic helminth and other parasite infections (47). Serum IgG4 concentrations also rise during allergy desensitization therapy, after repeated exposure to low doses of allergen (47). They have also been reported in the convalescent phase of the anti-viral response (35).

IgG4 antibodies do not fix complement and bind very poorly to activating FcγR (27), but they bind to the inhibitory FcγRIIB with an affinity that is higher than that of the other three IgG isotypes (27). Critical to the blocking activity of IgG4, inhibition is only mediated via the FcγRIIB receptor when immune complexes co-engage FcγRIIB and other activating FcRs (48). Despite high concentrations of specific antibodies of other isotypes, IgG4 should therefore block FcγR-mediated processes if it is present as a modest proportion of all antibodies in an immune complex. The high affinity of IgG4 should provide it with a competitive advantage, ensuring its participation in immune complex formation, and therefore allowing it to successfully act through FcγRIIB.

The ability of IgG4 antibodies to outcompete other isotypes may also be facilitated by the phenomenon of Fab arm exchange. In reducing conditions, IgG4 antibodies have the unique ability to dissociate into monovalent heavy/light chain pairs, and to re-associate again as bivalent antibodies (49, 50). This Fab arm exchange leads frequently to the formation of bi-specific antibodies. It has been suggested that this would, in practice, lead IgG4 antibodies to be functionally monovalent, as Fab arm exchange would be unlikely between antibodies of related specificities (49). We believe an alternative explanation of the consequences of Fab arm exchange could be the formation of blocking antibodies that bind with very high avidity because of their bi-specific nature.

Bivalency gives power to the IgG molecule. It most obviously allows a single antibody molecule to aggregate two antigen molecules, but it also allows high avidity binding to suitably spaced, repeated epitopes on the surface of a complex antigen. An additional outcome of bivalency has also been identified. It was first proposed on theoretical grounds that very weak binding of one arm of a bivalent antibody molecule to a “non-target” epitope could substantially improve the avidity of binding of the antibody to its target epitope, and that the probability of such bi-specific interactions was reasonably high (51). Recently, such bi-specific heteroligation was shown to facilitate antibody binding to HIV-gp140 (52). For a number of antibodies, high affinity interactions between gp140 and one antigen-binding site were supported by low affinity binding of the second antibody arm to completely different epitopes (52).

Fab arm exchange by IgG4 antibodies could improve the likelihood of heteroligation, if exchange occurs between antibodies of related specificities. This would be likely in two situations. IgG4 antibodies are undetectable in response to many antigens, and individuals with very low serum IgG4 concentrations are likely to have a limited IgG4 repertoire. In such circumstances, Fab arm exchange between antibodies targeting associated epitopes or related antigens would be more likely. In individuals with higher IgG4 concentrations, Fab arm exchange could also take place between antibodies of related specificities through their co-localization at sites of inflammation. At such sites, appropriate redox conditions of even a transient nature could lead Fab arm exchange to “lock together” Fab arms of associated specificities. This would function to increase IgG4 binding avidity, giving bi-specific IgG4 antibodies the ability to outcompete the more inflammatory isotypes, late in a response.

In addition to its IgG1-blocking activity, it is clear that IgG4 can block IgE-mediated immune function. The IgE and IgG4 isotypes are strongly linked with one another in the literature, and in fact IgE antibodies are often said to arise by class switching of IgG4^+^ B cells (53, 54). The mutational characteristics that we have reported clearly demonstrate that this is not always so, for both the number and patterns of mutations we have seen differ between IgE and IgG4 (11). However it is possible that IgG4 antibodies could be related to high affinity IgE, if such IgE antibodies arise late in a response. Clarification of this question, and determination of the circumstances in which such high affinity IgE might be produced by the human immune system will be necessary before late-arising IgE can be incorporated into the model.

The temporal model, as presented here, outlines sequential class switching during a first, persisting exposure to antigen. The nature of isotype expression in a recall response will clearly depend upon the tendency of class-switched cells to differentiate into memory cells during the primary response. Though IgM memory cells are now known to be an important part of the memory compartment (55, 56), there is some evidence from the early literature that IgG1 dominates the switched memory compartment. Studies of the recall response after re-challenge with keyhole limpet hemocyanin (KLH) showed little or no increase in peak concentrations of KLH-specific IgG2 and IgG3 antibodies, but a marked increase in circulating IgG1 anti-KLH antibodies (57). IgG4 antibodies were only seen upon re-challenge. The high affinity of IgG1 and its inflammatory functions make it the ideal isotype for IgG memory, and the logic of the Temporal Model suggests that memory cells of the other IgG isotypes could be either unhelpful or counterproductive.

The contributions that memory cells make to antibody isotype production in a recall response will depend upon whether memory cells re-enter germinal centers or immediately differentiate into plasmablasts and plasma cells. Studies in the mouse suggest that in a recall response, mouse IgG^+^ memory cells (55) and IgE^+^ memory cells (8) rapidly give rise to plasma cells, but IgM^+^ memory cells re-enter the germinal center reaction (55). If human and mouse cells are governed by similar processes, it may therefore be that events within the human germinal center during a recall response proceed as we have outlined for earlier events. In other words, reactivated IgM memory cells within the germinal centers would give rise to new IgE-switched and IgG-switched cells through a programed process of sequential switching.

Mechanisms that could underlie the temporal emergence of different IgG subclasses from the GC reaction will need to be explored, and one possibility lies in the recently reported competitive feedback between soluble antibody from plasma cells and the GC B cells (58). Soluble antibody, produced by cells that have previously emerged and differentiated, competes with GC B cells for binding to FDC-associated antigen. This competition promotes survival of GC B cells with higher affinity than the soluble antibody, while B cells of lesser affinity die by neglect. The Temporal Model suggests that as class switching proceeds, antibodies expressing subclasses from the more distally positioned IgG genes are likely to be of higher affinity. At a particular point in the response, higher affinity antibodies that express downstream IgG genes would outcompete soluble antibody of earlier subclasses, while promoting the destruction of B cells expressing earlier subclasses that carry fewer mutations and are of lower affinity. Feedback competition may therefore promote the temporal emergence of the subclasses in their genomic order.

Many studies give credence to the Temporal Model, but certainly this is not true of all studies. Discordant observations could be the result of pathogen-directed perturbations of normal immune function, for the temporal progression of isotype switching would be as susceptible as other aspects of immune function to subversion by bacterial and viral virulence factors (59). Discordant observations are particularly seen in some early studies of antibody isotypes, but these reports might be explained by the cross-reactivity of many early “isotype-specific” reagents (60). Others might now be explained by phenomena such as Fc–Fc binding of IgG4 antibodies that were unknown until recently (50). But the resolution of the mystery of antibody function cannot come from studies of the past. It is our hope that this description of the Temporal Model will encourage the question of antibody isotype function to be revived. Having received so little attention over the last two decades, it is now time for the power of high throughput sequencing to be harnessed, to confirm the relationship between the levels of mutation and antibody isotypes in individuals of different ethnicities and states of health, and to properly address the clonal relationships between B cells producing antibodies of different isotypes. It may then be that the timing of class switching, the passage of different cell populations between anatomical compartments within the lymph node, the emergence of cells from the germinal center reaction, and the overall functions of human isotypes can finally be determined with certainty.

## Conflict of Interest Statement

The authors declare that the research was conducted in the absence of any commercial or financial relationships that could be construed as a potential conflict of interest.
